# A novel stop codon mutation in exon 5 (c.639G>A) of the cadherin-1 gene in a Vietnamese man with hereditary diffuse gastric cancer: a case report

**DOI:** 10.1186/s13256-021-02837-y

**Published:** 2021-05-04

**Authors:** Dzung Ngoc Thi Dang, Huong Thanh Thi Nguyen, Hoa Dieu Ngo, Bac Manh Tran, Anh Duc Vu, Huy Quang Dang, Van Thanh Ta

**Affiliations:** 1 Biochemistry Department, Hanoi Medical University, Hanoi, Vietnam; 2 Department of Functional Exploration, National Geriatric Hospital, Hanoi, Vietnam; 3 Department of Medical Laboratory Science, Faculty of Medical Technology, Hanoi Medical University, Hanoi, Vietnam

**Keywords:** Case report, Cadherin-1, Hereditary diffuse gastric cancer, C.639G>A

## Abstract

**Background:**

Germline pathogenic variants in the cadherin-1 (*CDH1*) gene cause a predisposition to hereditary diffuse gastric cancer (HDGC). We report an HDGC case in Vietnam and identify a novel mutation in the *CDH1* gene.

**Case presentation:**

A 28-year-old Vietnamese man was diagnosed with HDGC and a novel mutation at c.639G>A. All exons of *CDH1* were sequenced in his pedigree, which revealed the c.639G>A mutation in the proband, his father, and uncle. The patient refused treatment and died 4 months after diagnosis. Endoscopic surveillance of the father and the uncle showed structural abnormalities in the father.

**Conclusion:**

In cases of HDGC, identification of the *CDH1* gene mutation is very important for better counseling and more effective strategies to prevent the development of diseases, such as prophylactic gastrectomy for family members with genetic mutations.

## Background

Hereditary diffuse gastric cancer (HDGC) is a diffuse-type autosomal dominant familial syndrome caused by a germline mutation in the cadherin-1 gene (*CDH1*) [1]. The *CDH1* gene is located on chromosome 16q22.1 and is composed of 16 exons that transcribe the E-cadherin protein [2]. The syndrome is associated with a lifetime risk for mutation carriers of 70% in men and 56% for women, and a 42% risk for lobular breast cancer (LBC) in women [3]. Patients with gastric cancer and germline *CDH1* mutations have a lower survival rate than patients who meet the HDGC criteria but do not have the *CDH1* mutations [4]. Early detection of HDGC is also challenging because early-stage signet ring cell carcinoma is difficult to visualize using gastroduodenoscopy with random biopsies [5]. Therefore, the follow-up and management of carriers of a pathogenic germline *CDH1* mutation, especially in the family proband, is necessary to prevent their death from invasive carcinoma [6]. To date, there has been no report of *CDH1* or its mutation in Vietnamese patients. In this report, we present a novel c.639G>A (p.W213*) mutation in a Vietnamese man with HDGC.

## Case presentation

In September 2016, a 28-year-old Vietnamese male patient visited the National Geriatric Hospital in Hanoi due to epigastric pain, weight loss, and painful swollen lymph nodes in his neck for the past 5 months. He had no history of gastric disease. The subsequent workup included a gastroscopy that revealed an ulcer in the body of the stomach measuring more than 3 cm in diameter. The biopsy from gastroscopy was assessed for *Helicobacter pylori* by the *Campylobacter*-like organism (CLO) test and histopathological examination. Histopathology results for the ulcer were negative for *H. pylori* and revealed stage IV diffuse-type gastric cancer (DGC), characterized by signet ring cells. Unfortunately, for financial reasons, the patient refused additional diagnosis including neck lymph node biopsy, computed tomography (CT) scan, and positron emission tomography (PET)/CT to evaluate distant metastasis, and also refused to follow up on further treatments. The patient's health deteriorated, and he died 4 months post-diagnosis.

The patient’s family history did not show DGC. However, because of the clinicopathological presentation including early onset, severe clinical symptoms, and histopathological test results showing diffuse-type characteristics, we performed genetic screening for mutations in the *CDH1* gene by E-cadherin protein expression.

With the patient’s consent, we used the Exgene™ Blood SV kit (GeneAll Biotechnology, Korea) to extract DNA from the whole blood sample according to the instruction manual. This was followed by polymerase chain reaction (PCR) amplification and DNA sequencing using both forward and reverse primers for all exons and introns of the *CDH1* gene. The primers were designed using National Center for Biotechnology Information (NCBI) Primer-BLAST [Basic Local Alignment Search Tool] and the QIAGEN CLC Main Workbench (version 8.1.2.) and were reanalyzed using the OligoAnalyzer tool. After the polymerase chain reaction (PCR), we separated the DNA by size using gel electrophoresis (1.5% agarose gel). Finally, we performed gel analysis using the UVP GelDoc-It^2^ Imaging System (Fisher Scientific). The determination of DNA sequences for all samples was done on Applied Biosystems ABI 3500 (JP0 and ABI 3730xl; Thermo Fisher) deoxyribonuclease acid (DNA) analyzers, followed by gene analysis using BioEdit (version 2.0) and ApE (version 1.0) software.

**Fig. 1:**
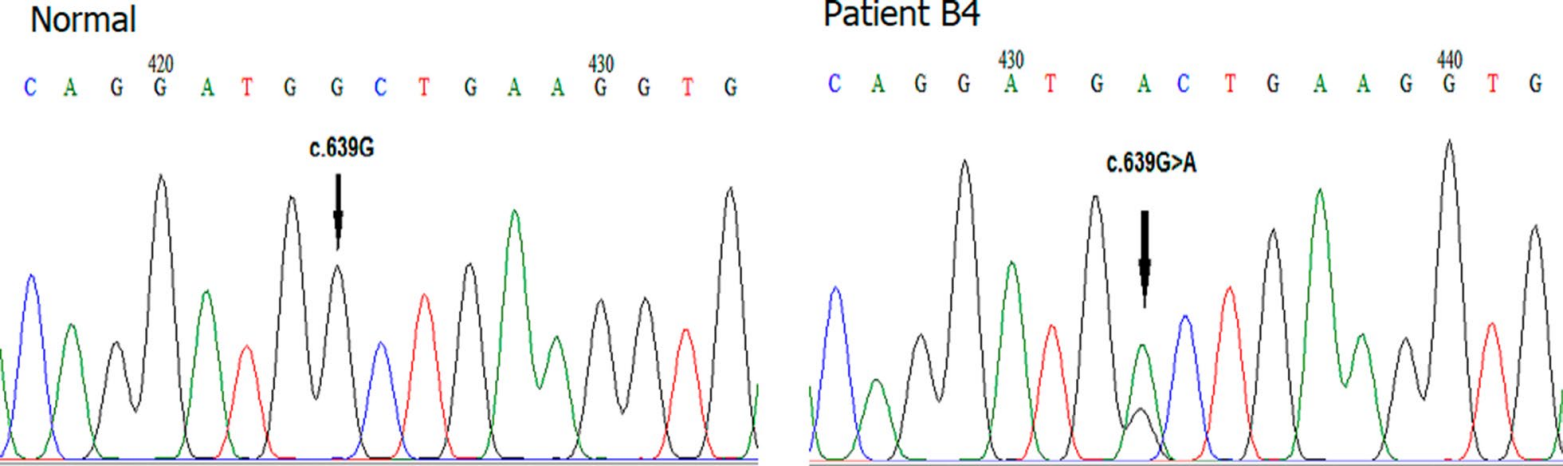
Analysis of exon 5 deoxyribonucleic acid sequencing; c.639G>A, p.W213* mutation was located. Patient B4 was the proband

**Fig. 2:**
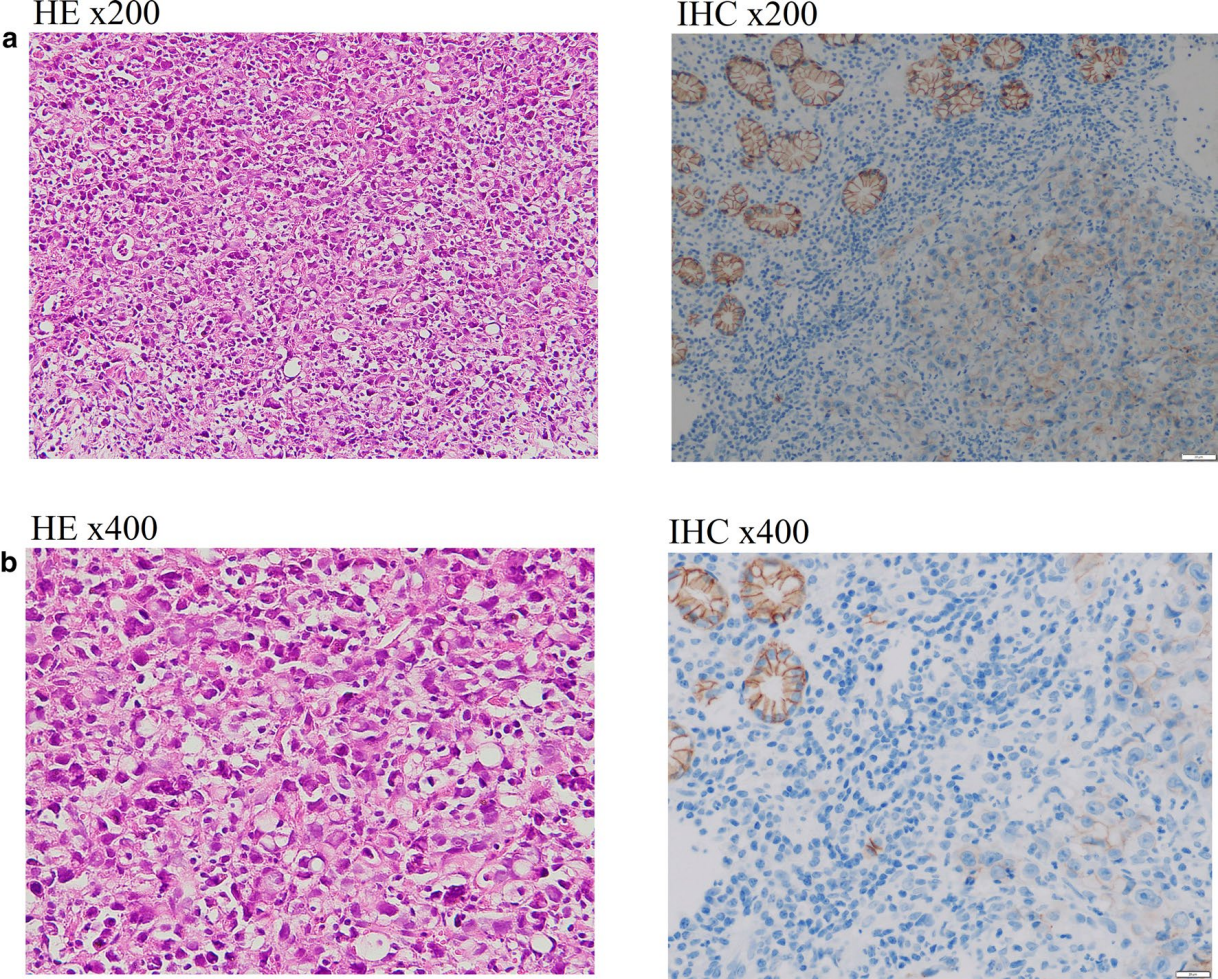
Histopathology staining from the biopsy of the patient's stomach through gastroscopy. **a** Hematoxylin and eosin (H&E) staining with poorly cohesive carcinoma, characterized by signet ring cells. The distribution of cells is diffuse; they can form small groups of bands of gland-like structures with a conspicuous desmoplastic reaction of the stroma. **b** Immunohistochemical staining showed decreased E-cadherin expression. Positive staining is shown in brown. Representative images of E-cadherin staining demonstrated lower expression of E-cadherin

We found a novel heterozygous nonsense mutation that resulted in a premature termination codon (PTC) UGA at p.213 in exon 5 (*CDH1* c.639G>A, p.W213) (Fig. 1). The mutation shortened the *CDH1* gene length from 882 aa to 213 aa. The immunohistochemical staining of the patient’s tissue sample showed decreased expression of E-cadherin (Fig. 2).

**Fig. 3:**
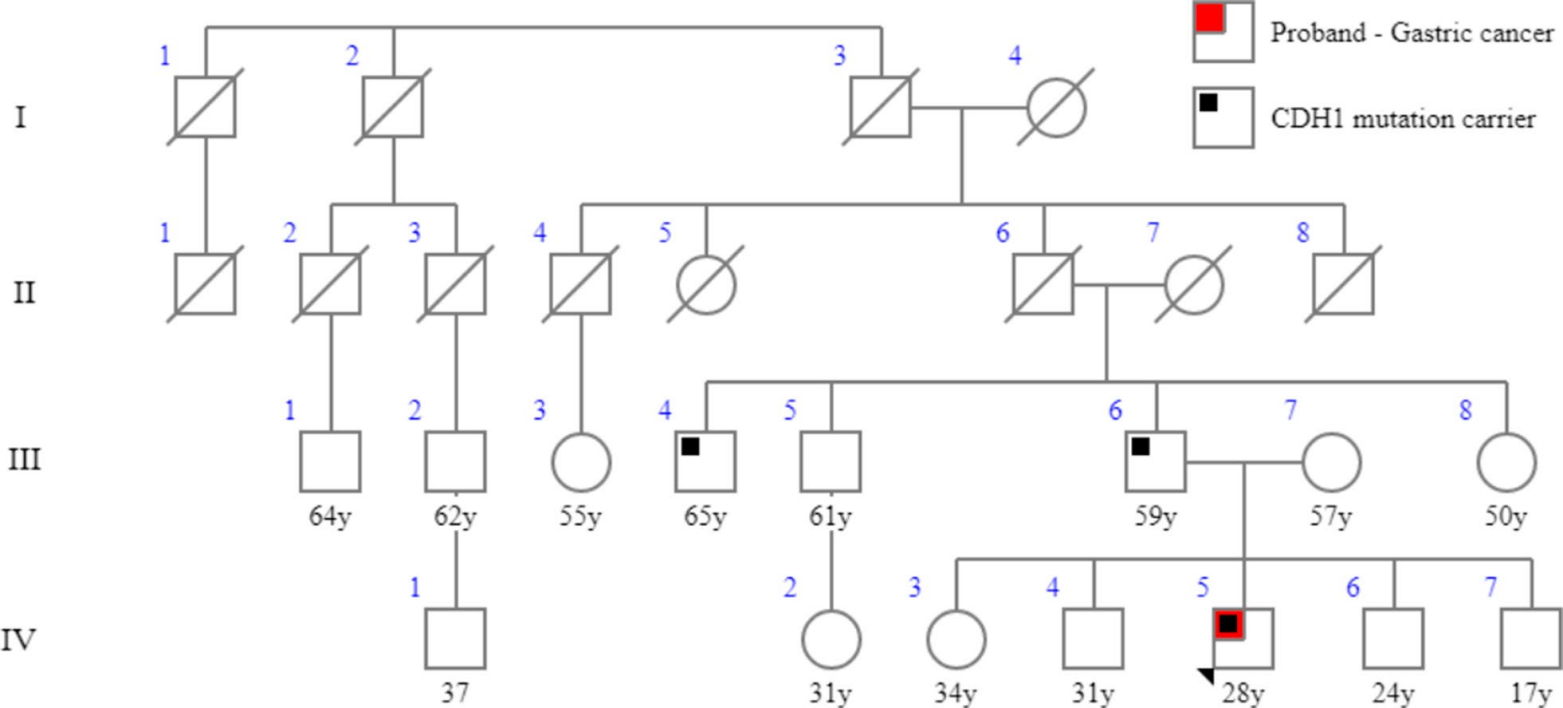
Patient’s pedigree showing two members of the family carrying the cadherin-1 c.639G>A mutation

We collected blood samples from the patient’s family members (11 participants) for mutation screening in the *CDH1* gene exon 5. The participants included three first cousins once removed (III-1, III-2, III-3), two uncles (III-4, III-5), the father (III-6), the mother (III-7), a male first cousin (IV-1), a female first cousin (IV-2), an older sister (IV-3), and a younger brother (IV-7) (Fig. 3). At the time of testing, no participants showed any symptoms of HDGC. Upon analysis of the pedigree, we found the patient’s parents to be healthy, and there was no family history of the disease. The DNA sequencing in *CDH1* exon 5 showed a similar mutation (c.639G>A, p.W213*) in two members of the family: the father (III-6, 59 years old) and an uncle (III-4, 65 years old), with both heterozygous. Gastroscopy performed on the patient’s father revealed inflammation and antral gastritis. Six-core biopsy specimens showed chronic gastritis, intestinal metaplasia, and low-grade dysplasia. High iron diamine staining for metaplasia typing was negative.

## Discussion and conclusions

The reported patient was diagnosed with HDGC stage IV at a young age, characterized by severe clinical manifestations, which matched the latest guideline for diagnosis of HDGC [7]. The novel c.639G>A nonsense mutation reduced the expression of the E-cadherin protein due to the premature stop codon in the coding region of exon 5. Enrique and colleagues reported a heterozygous mutation c.1531C>T (p.Gln511*) which caused a PTC in a 22-year-old male patient with HDGC stage IV. Similar to the present case, the patient’s family history showed no sign of HDGC, and he died shortly after the diagnosis [8]. Another novel *CDH1* mutation (c.1612delG), discovered by Caggiari and colleagues in 2017, also resulted in a PTC at codon 556 (p.Asp538Thrfs*19) in the extracellular fourth repeat region of a 41-year-old man with HDGC. In this case, the disease progressed rapidly to peritoneal, pancreatic, and hepatic metastasis despite 3 months of targeted therapy [9]. Both studies demonstrated that *CDH1* nonsense mutations are associated with more severe clinical presentation and poor prognosis in patients with early-onset HDGC.

While none of the patient’s relatives had HDGC, we found the same *CDH1* c.639G>A mutation in the tissue of the patient’s father and uncle, who were both elderly. The results from six-core biopsy and gastroscopy of the father showed the absence of cancer cells. The mechanism behind the formation of signet ring cell carcinoma in carriers of the c.639G>A mutation is unclear; however, we speculate that environmental factors may play a role in the process.

In recent years, HDGC incidence has been increasing in Asia, the United States, and Europe, despite being a rare inherited condition [10]. The rapid disease progression, difficulty in early diagnosis, and highly metastatic nature contribute to the poor prognosis, especially in patients with early-onset disease. A study by van der Post *et al.* in 2015 showed a lower survival rate for gastric cancer patients with a *CDH1* mutation compared to non-carriers, with 5-year survival rates of 4% and 13%, respectively [4]. There are several problems regarding the diagnosis of gastric cancer such as difficulty in detecting cancer tissue in the gastric mucosa or even after biopsy. This indicates the need for total surveillance of individuals carrying a *CDH1* mutation, especially those with a family history of gastric cancer. This includes periodic checkups or prophylactic total gastrectomy (PTG), which is recommended for *CDH1* mutation carriers. In a systematic review of the histopathological diagnosis of gastric cancer, 87% of cases were detected from prophylactic gastrectomy [8, 11]. However, postoperative complications following PTG such as diarrhea, fatigue, acid reflux, and internal bleeding should be anticipated. Furthermore, considering the uniqueness of the healthcare system and the mindset of Vietnamese people, providing explanations and counseling to *CDH1* carriers regarding PTG has proven to be challenging, mainly because of the absence of disease and/or symptoms. Thus, further research is needed to fully elucidate the effects of PTG in people with *CDH1* mutations and those with family members having HDGC.

The limitation of the study was that the patient did not attend treatment after being diagnosed with HDGC. Therefore, we did not have the opportunity to evaluate and classify the disease stages according to the TNM Classification of Malignant Tumors and could not evaluate the response to therapy. As a result, we were unable to thoroughly analyze the relationship between the patient's clinical, subclinical, and genetic changes.

Case study research in Vietnam concerning *CDH1* mutations in stomach cancer is rare, and there is a lack of emphasis on genetic screening in HDGC diagnosis. Early identification of *CDH1* mutations in HDGC patients and their family members is a significant factor enabling better counseling and preventive strategies such as PTG against the disease in mutation carriers. More importantly, it provides recommendations for the development of disease prevention strategies and formulation of more specific governmental health policies to support the treatment of Vietnamese patients in extraordinary circumstances in the future.

## Data Availability

The datasets used and/or analyzed during the current study are available from the corresponding author on reasonable request.

## Abbreviations

HDGC: Hereditary diffuse gastric cancer
DGC: Diffuse-type gastric cancer
CDH1: Cadherin-1
PTG: Prophylactic total gastrectomy
PTC: Premature termination codon
